# Effects of Robot-Assisted Training for the Unaffected Arm in Patients with Hemiparetic Cerebral Palsy: A Proof-of-Concept Pilot Study

**DOI:** 10.1155/2017/8349242

**Published:** 2017-07-06

**Authors:** Alessandro Picelli, Elisabetta La Marchina, Antonella Vangelista, Elena Chemello, Angela Modenese, Marialuisa Gandolfi, Elisa Francesca Maria Ciceri, Alessandra Bucci, Giada Zoccatelli, Leopold Saltuari, Andreas Waldner, Alessio Baricich, Andrea Santamato, Nicola Smania

**Affiliations:** ^1^Neuromotor and Cognitive Rehabilitation Research Center, Department of Neurosciences, Biomedicine and Movement Sciences, University of Verona, Verona, Italy; ^2^Polyfunctional Center “Don Calabria”, Verona, Italy; ^3^Neurorehabilitation Unit, Department of Neurosciences, Hospital Trust of Verona, Verona, Italy; ^4^Neuroradiology Unit, Department of Neurosciences, Hospital Trust of Verona, Verona, Italy; ^5^Department of Neurology, Hochzirl Hospital, Zirl, Austria; ^6^Research Unit of Neurorehabilitation, Bolzano, South Tyrol, Italy; ^7^Villa Melitta Rehabilitation Clinic, Bolzano, Italy; ^8^Health Sciences Department, Università del Piemonte Orientale, Novara, Italy; ^9^Physical Medicine & Rehabilitation Section, “OORR” Hospital, University of Foggia, Foggia, Italy

## Abstract

On a voluntary basis, 10 adolescents with hemiparesis due to cerebral palsy and 11 neurologically healthy control subjects participated in this proof-of-concept pilot study. The aim was to examine the effects of robot-assisted training for the unaffected arm in patients with hemiparetic cerebral palsy. Baseline comparison between the unaffected arm of the hemiparetic patients with cerebral palsy and the dominant arm of healthy control subjects showed significant differences on the Jebsen-Taylor Hand Function test and action planning ability tests. Within-group comparison after ten 30-minute sessions (five days a week for two consecutive weeks) of robot-assisted training for the unaffected arm showed significant improvements in patients with cerebral palsy on the Jebsen-Taylor Hand Function test (performed at both hands) and action planning ability test (evaluated at the unaffected arm). Our findings are in line with previous evidences of action planning deficits at the unaffected arm in patients with hemiparetic cerebral palsy and support the hypothesis that robot-assisted training for the unaffected arm may be useful to improve manual dexterity and action planning in patients with hemiparesis due to cerebral palsy.

## 1. Introduction

Cerebral palsy (CP) is the most common condition of all childhood disabilities, affecting about 2.0–3.5 live births out of 1000 in the United States [1]. It encompasses a heterogeneous group of neurodevelopmental conditions that primarily present as disorders of movement and posture, often accompanied by epilepsy, secondary musculoskeletal problems, and impaired sensation and cognition [1, 2]. By definition, CP results from abnormal brain development and/or brain damage that is nonprogressive and occurs during very early development [1–3]. Symptom onset occurs during early childhood (typically before 18 months of age, with diagnosis confirmed, on average, at 13–19 months) [3].

The most common form of this condition is hemiparetic CP, which alters use of one hand by impairing contralesional motor output [3–8]. Patients with hemiparetic CP may experience difficulty in executing movements at the affected arm, such as increased number of submovements, stereotyped shoulder-elbow recruitment order, and variability of hand trajectories, with movement patterns characterized by the application of inappropriately coordinated grip and lift forces [7, 8]. Current research suggests that motor impairments in patients with hemiparetic CP result from damage to the corticospinal tract, as well as from impairments of sensorimotor pathways and motor action planning [3].

The main current approaches to upper limb rehabilitation in patients with hemiparetic CP are modified constraint-induced movement therapy and bimanual intensive therapy for improving movement execution deficits mainly through motor learning concepts [3, 9, 10]. Robot-assisted training is an emerging modality for CP rehabilitation that uses robotics to aid movement of the limbs during exercises [3]. It allows participants to experience a large amount of repetitive, goal-directed, movements in association with sensory feedback in an attractive environment, which are necessary for improving motor function in patients with CP [11–17]. As to its mechanism of action, robot-assisted upper limb practice has been shown to facilitate motor skill acquisition through enhanced sensorimotor control by improving motor planning processes [18]. This is in accordance with previous literature reporting that robot-guided practice of upper limb reaching movements primarily influences motor planning rather than online motor control mechanisms [18].

Motor planning is fundamental to obtaining optimal task performance and selecting the most appropriate movement strategy [4]. It refers to the ability to anticipate the upcoming action when preparing a movement towards an object (e.g., to pick up objects smoothly, planning of grasping and manipulation is necessary as the object's weight and center of mass can only be determined after it has been lifted) [11, 19]. In particular, when something is grasped, the selection of an appropriate grip is critically dependent on the subsequent action that needs to be performed with the object [19]. The planning of forces is thought to be based on internal models of the object's physical properties gained during previous manipulation with the same objects [11, 19]. Impaired anticipatory control in CP is probably due to an altered internal representation of the movement as a result of the limited ability to integrate sensory information with motor output [11]. This notion is further confirmed by the growing evidence for the presence of anticipatory planning deficits also at the unaffected upper limb of patients with hemiparetic CP [4, 7, 8, 19, 20].

Children with hemiparetic CP are noted to more appropriately plan fingertip forces when lifting an object with their affected hand after performing several lifts with the unaffected hand immediately before [21, 22]. This suggests that object information is transferred in a feedforward fashion from the unaffected to the affected hand in children with hemiparetic CP [11, 23]. On this basis, and according to the concepts described so far, there is a rationale for the application of robot-assisted training on the unaffected arm to improve motor action planning and reduce sensorimotor impairment in patients with hemiparetic CP [23]. The aim of the present study was to obtain proof-of-concept of these hypotheses by examining the effects of robot-assisted training for the unaffected arm in patients with hemiparetic CP.

## 2. Methods

On a voluntary basis, 10 patients with hemiparesis due to CP (4 with left hemiparesis and 6 with right hemiparesis; mean age, 14.5 years) and 11 neurologically healthy control subjects (all right-handed; mean age, 14.2 years) participated in this single-center proof-of-concept pilot study. To characterize patients with hemiparetic CP in terms of hand function, we used the Manual Ability Classification System (MACS) that describes five levels of ability to handle objects in daily activities: level I—handles objects easily and successfully; level II—handles most objects but with a somewhat reduced quality and/or speed of achievement; level III—handles objects with difficulty and needs help to prepare and/or modify activities; level IV—handles a limited selection of easily managed objects in adapted situations; level V—does not handle objects and has severely limited ability to perform even simple actions [24, 25]. The characteristics of sample are presented in Table 1.

The parents of patients and control subjects provided signed informed consent for participation in the study. The study was carried out according to the Declaration of Helsinki and approved by the local ethics committee of our institution. During the study period, patients were asked to refrain from engaging in any form of physical therapy or home exercise program other than that scheduled in the study protocol.

### 2.1. Treatment Procedures

After baseline evaluation, all patients with hemiparetic CP took part in a robot-assisted training program for the unaffected arm consisting of ten 30-minute sessions, 5 days a week (from Monday to Friday), for 2 consecutive weeks. Robot-assisted arm training was carried out on an ARMEO® Spring System (Hocoma AG, Volketswil, Switzerland), which is a spring-instrumented exoskeleton with seven degrees of freedom and one pressure sensor [17]. Springs provide passive arm weight support and guidance (stiffness can be adjusted to different levels of gravity support and muscular involvement, enabling subjects to achieve a large range of motion within a three-dimensional workspace with their own residual functionality). The ARMEO exoskeleton can be adapted to the patient's morphology by changing the position and length of the orthosis (we used a pediatric version of the device). A pressure-sensitive handgrip allows for grasp training.

The patients with CP performed sets of exercises under the supervision of a physiotherapist. The exercises were individualized to the needs of each patient and selected to provide an engaging and gradual training experience with increasing difficulty. Each 30-minute training session was divided in two parts: 15 minutes of passive exoskeleton training and 15 minutes of task-oriented exercises based on reaching, manipulation, grip selection, grasping, and lifting activities. During passive exoskeleton training, the exercises involved different joints of the unaffected arm (the shoulder, elbow, and wrist) with predetermined (separated or combined) movements (flexion/extension, abduction/adduction, and pronation/supination) performed in a one-dimensional, two-dimensional, or three-dimensional environment with increasing demand on accuracy or speed. As to the “virtual” task-oriented exercises, the patients performed functional tasks of increasing difficulty that involved several different activities performed with their unaffected arm, such as breaking eggs, cleaning a surface, posting a letter, or watering flowers [17].

### 2.2. Evaluation Procedures

Patients with hemiparetic CP were evaluated before (T0) and immediately after treatment (T1). Healthy control subjects were evaluated only at baseline. The same rater evaluated all participants.

#### 2.2.1. Outcome Measures

The Nine-Hole Peg test (NHPT) assesses hand dexterity. It requires taking 9 pegs from a container and placing them into 9 holes on a board and vice versa as quickly as possible [26–28]. Both arms of patients with hemiparetic CP and healthy controls were tested. The score was the time taken to complete the test activity [26–28].

The Jebsen-Taylor Hand Function test (JTHF) is a standardized test for assessing a person's overall hand function. It consists of seven subtests (writing sentences, simulated page turning, lifting small objects, simulated feeding, stacking checkers, lifting large light objects, and lifting large heavy objects) that simulate activities of daily living [29, 30]. Both arms of the patients with hemiparetic CP and the healthy controls were tested. The score was the time taken to complete fine motor, gross motor, nonweighted, and weighted tasks measured with a stopwatch [29, 30].

**Figure 1:**
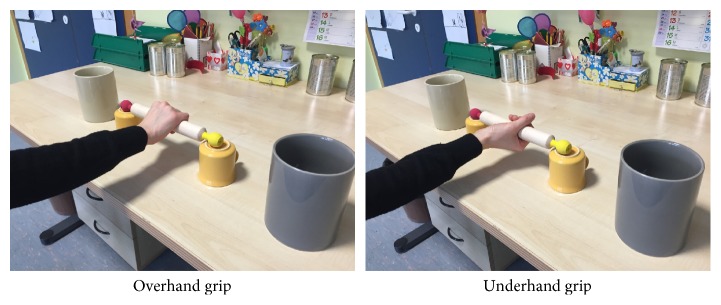
Setup of the Stick Test for evaluating action planning.

Action planning was evaluated at the unaffected hand of patients with hemiparetic CP and the dominant hand of healthy controls as described in the literature [31–35]. The first test (Stick Test) uses a wooden pin (28 cm long, 3 cm in diameter, and approximately 100 g) [34]. One end of the pin is painted yellow and the other end red. The pin rests horizontal on two supports 7 cm above the table in front of the subject. The yellow end of the pin points to the right and the red end points to the left from the participant's perspective. A grey cup is placed near the yellow end of the pin and a white cup near the red end (see Figure 1() for details of apparatus setup).

Each participant was asked to simply pick up the pin with one hand while leaving the other hand resting on the knee and without manipulating the pin once grasped. There were four types of trials (placing the yellow end into the grey cup; the yellow end into the white cup; the red end into the grey cup; and the red end into the white cup) presented in random order. There were five blocks, for a total of 20 trials. The score was the percentage of starting grips that were consistent with the end-state comfort concept (people using their right hand to perform the test use an underhand grip to place the left red end of the pin into either the white or the grey cup and an overhand grip to place the right end of the pin into either the white or the grey cup; people using their left hand to perform the test use an overhand grip to place the left red end of the pin into either the white or the grey cup and an underhand grip to place the right end of the pin into either the white or the grey cup) [34].

**Figure 2:**
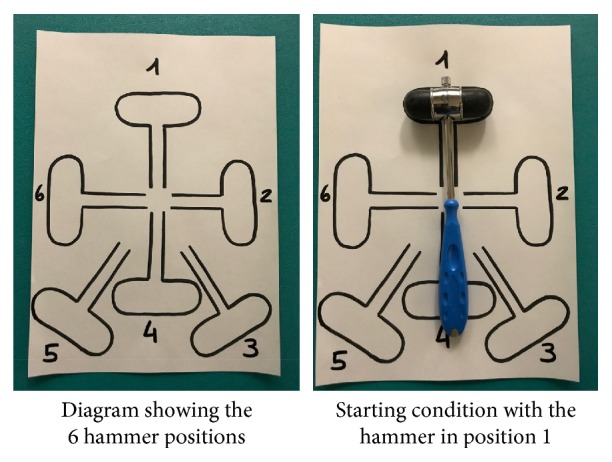
Setup of the Hammer Test for evaluating action planning.

The second test (Hammer Test) uses a metal medical hammer (22 cm long, handle 2 cm in diameter, and approximately 120 g) [35] placed on a table and next to a sheet of paper (30 cm × 28 cm) with the outlines of 6 hammer rotations (see Figure 2() for details).

The Hammer Test always started from a condition that did not require any hammer rotation (position 1). Each participant was asked to pick up the hammer and pound the table. After successful performance, the participant was asked to repeat the task, but with the hammer placed in a different starting position (positions from 2 to 6). Rotation from the starting position of the hammer was repeated three times in random order, resulting in a total of 18 trials per subject. Performance was scored according to whether the hand posture at the end of the action was comfortable (i.e., with the thumb pointing towards the end goal) or uncomfortable (i.e., with the thumb pointing opposite the end goal). For the analyses, we distinguished between critical conditions (where an uncomfortable starting posture was needed to allow a comfortable end posture) and control conditions (where a comfortable starting posture resulted in a comfortable end posture). For the critical conditions, orientations 2 and 3 were used for the right-handed and orientations 5 and 6 for the left-handed. The remaining orientations were regarded as control conditions (orientations 1, 2, 3, and 4 for the left-handed; orientations 1, 4, 5, and 6 for the right handed). The percentage of comfortable end postures in the critical condition was analyzed [34].

### 2.3. Statistical Analysis

Statistical analysis was carried out using the Statistical Package for Social Science for Macintosh, version 20.0 (IBM Corp., Armonk, NY, USA). The Mann–Whitney *U* test was used to compare patients with hemiparetic CP versus healthy control subjects as to their performance in all outcomes at baseline. Wilcoxon signed-rank test was used to perform after versus before treatment within-group comparisons for all outcomes in patients with hemiparetic CP. In order to evaluate the presence of “learning effects” of the two motor planning ability tests, we used the Wilcoxon signed-rank test to compare the last set of trials versus the first one at baseline evaluation. The alpha level for significance was set at *P* < 0.05.

## 3. Results

Baseline comparison between patients with hemiparetic CP and healthy control subjects (the unaffected arm versus the dominant arm) showed significant differences on the JTHF (*P* = 0.015; *Z* = −2.433), the Stick Test (*P* = 0.003; *Z* = −2.932), and the Hammer Test (*P* = 0.003; *Z* = −2.990) for action planning. Conversely, no significant difference was found on the NHPT (*P* = 0.104; *Z* = −1.628). Furthermore, no significant differences were found on the Stick Test (*P* = 0.180; *Z* = −1.342) and the Hammer Test (*P* = 0.191; *Z* = −1.307) when the last set of trials was compared against the first set for evaluating the presence of “learning effects” (Table 2).

Posttreatment versus baseline comparison among patients with hemiparetic CP showed significant improvements on the JTHF at both the affected (*P* = 0.028; *Z* = −2.201) and the unaffected (*P* = 0.028; *Z* = −2.201) arms, as well as on the Stick Test (*P* = 0.034; *Z* = −2.121) and the Hammer Test (*P* = 0.042; *Z* = −2.032) for action planning at the unaffected arm. Conversely, no significant improvement was found on the NHPT for both the affected (*P* = 0.854; *Z* = −0.184) and the unaffected (*P* = 0.345; *Z* = −0.944) arms (Table 2).

## 4. Discussion

A fundamental aspect of motor control is action planning, which can be defined as the ability to take upcoming task demands into account when first taking hold of an object [4]. This strategy requires a feedforward mechanism based on an internal image of the object's characteristics [23]. At about the age of 2 years, children learn to use an internal model and continue to refine their strategy with age. By about the age of 8 years, the strategy is essentially the same as that of adults in children able to update their internal image of the object when its properties are changed [23, 34, 36].

Action planning implies that people plan the end of an action based on the end-state comfort effect. This means that people most often choose to terminate a movement in a comfortable position even if it requires taking an uncomfortable initial posture (i.e., to turn over an upside-down cup, people will initially grasp it in an uncomfortable posture, so that the arm is in a comfortable posture when the cup is turned over at the end of the task) [4, 7, 8, 23, 35, 37]. Since motor programs can be selected for each limb by the contralateral hemisphere, previous studies have suggested that the left hemisphere may be the planning central system that integrates movements and monitors coordination, given that the right hand is dominant in the majority of the population [4, 38]. An alternative account of motor planning proposes a more distributed system across both hemispheres based on the evidence for distinct bilateral and contralateral activations in the precentral gyrus during movement, with the former thought to reflect motor planning and the latter motor execution [39].

Patients with CP have been noted not to use the full feedforward sensorimotor coordination strategy for manual dexterity that healthy people use; instead, they employ a slower strategy with elements adopted by healthy children aged less than 2 years [23]. A probable consequence of the impaired anticipatory control due to an altered internal model of movement representation [11] is that people with hemiparetic CP show anticipatory planning deficits involving also the unaffected upper limb (according to the selection of an initial grip that ensures a comfortable posture at the start of a movement sequence instead of optimizing comfort of the end posture as is frequently seen in healthy subjects) [4, 7, 8, 19, 20, 32, 33]. Interestingly, patients with CP are able to develop anticipatory adjustments in their motor control strategy with longer practice [22, 23, 40]. Furthermore, learning of anticipatory strategies acquired with the unaffected hand might be transferred and used during movements of the affected hand [22, 40].

Along this line, the present pilot study provides a proof-of-concept for the potential usefulness of robot-assisted training for the unaffected arm to improve manual dexterity and action planning in patients with hemiparetic CP. Before discussing our results, however, we wish to point out that the motor planning ability evaluation tests we used in this pilot study have not been validated in patients with CP and that potential “learning effects” due to tests repetition cannot be excluded. So the strength of our findings needs to be interpreted with caution. That said, the patients with hemiparetic CP participating in this pilot study showed some deficits in manual dexterity and anticipatory planning at the unaffected arm when compared to the dominant upper limb of the healthy control subjects at baseline. Our findings are shared by previous work on action planning deficits in hemiparetic CP and on the absence of motor planning lateralization in children with hemiplegia, given that we enrolled CP patients with left or right hemiparesis [4]. Furthermore, we observed significant improvements in manual dexterity and anticipatory planning in patients with hemiparetic CP after 10 sessions of robot-assisted training for the unaffected arm. These observations are consistent with the notion that training of the unaffected arm may be useful to improve feedforward strategies of sensorimotor coordination based on internal representations of external objects in patients with hemiparetic CP [22, 23, 40]. Moreover, since repetition over time is needed before action planning improvements can be seen in patients with CP [11, 21–33, 40], this can be advantageously provided with robot-assisted arm training, as reported in previous studies on CP [16, 17].

Developmental and postlesional (re)organizational issues of the human central nervous system about hand motor control might be mentioned to explain our results [41–45]. In patients with minor brain lesions, the corticospinal tract of the affected hemisphere still allows for exerting sufficient motor control over the contralesional hand. Conversely, patients with lesions disrupting the corticospinal tract of the affected hemisphere show severe motor impairment owing to the presence of abnormal ipsilateral corticospinal projections to the affected hand. Also, patients with partial integrity of the crossed corticospinal tract and the presence of abnormal ipsilateral projection have bilateral projections to the affected hand with alternative pathways from both hemispheres to control it [41, 44].

Unfortunately, because of its pilot nature, we cannot infer anything about the functional integrity of crossed corticospinal tracts or the presence of abnormal ipsilateral projections to the affected hand in our sample, as we did not perform neurophysiological or functional neuroimaging evaluations. This is only one of the several limitations besides the small sample size, the lack of a control group for robot-assisted training, and the absence of follow-up evaluations. Because of the small sample size, our study is underpowered to evaluate the role of other factors that may have contributed to the differences observed between the unaffected arm of the CP patients and the healthy controls, such as the level of impairment as measured by the MACS. Furthermore, though no significant differences were found on the action planning tests when we compared the last set of trials versus the first one to check for the presence of “learning effects”, we had no control group to repeat the tests at a later time and compare scores versus the subjects that had received robot-assisted arm training. Therefore, we cannot exclude the possibility that the posttreatment changes in test scores were actually due to the training effects and not to repeated exposure to the tests. Finally, as mentioned above, we used two tests (the Stick and the Hammer Tests) that have not been validated for evaluating motor planning ability. Nonetheless, to the best of our knowledge, there is currently no motor planning ability evaluation test validated for use in patients with CP. In future studies on this population, validated tests to measure outcomes are needed in order to compare the effects of a physical intervention on motor planning ability.

## 5. Conclusions

Our findings are in line with previous work on action planning deficits in patients with hemiparetic CP and support the hypothesis that robot-assisted training for the unaffected arm may be useful to improve manual dexterity and action planning in patients with hemiparesis due to CP. Nevertheless, it should be emphasized that the strength of our conclusions is very limited. Blinded, randomized controlled trials involving a larger sample are needed to overcome the limitations of this proof-of-concept pilot study and evaluate the role of unaffected arm training in hemiparetic CP rehabilitation.

## Figures and Tables

**Table 1:** Characteristics of patients and healthy controls.

Patients with hemiparetic CP	Age (years)	Gender	Affected hand	MACS level
1	15.3	Female	Left	I
2	10.8	Female	Right	II
3	14.8	Male	Right	II
4	17.3	Male	Left	III
5	15.8	Male	Left	I
6	10.9	Female	Right	I
7	13.1	Male	Left	II
8	16.3	Male	Right	I
9	12.3	Male	Right	I
10	16.4	Male	Right	I

Healthy controls	Age (years)	Gender	Dominant hand	

1	15.3	Female	Right	
2	17.9	Male	Right	
3	11.1	Female	Right	
4	13.6	Male	Right	
5	11.1	Male	Right	
6	14.1	Male	Right	
7	15.9	Female	Right	
8	10.7	Male	Right	
9	16.5	Female	Right	
10	16.1	Female	Right	
11	14.7	Male	Right	

CP: cerebral palsy; MACS: manual ability classification system.

**Table 2:** Performance on outcome measures.

Outcome		Participants	Hand	Baseline	Between-group comparisons at baseline (unaffected versus dominant)	After treatment	Within-group comparisons (after treatment versus baseline)
Nine-Hole Peg Test (s) Mean (SD)		CP patients	Affected	31.5 (4.3)		32.3 (10.6)	*P* = 0.854 (*Z* = −0.184)
			Unaffected	13.8 (4.6)	*P* = 0.104 (*Z* = −1.628)	12.6 (1.2)	*P* = 0.345 (*Z* = −0.944)
		Healthy controls	Dominant	11.5 (1.3)			
			Nondominant	12.1 (1.1)			

Jebsen-Taylor Hand Function test (s) Mean (SD)		CP patients	Affected	17.9 (13.3)		14.5 (6.9)	*P* = 0.028 (*Z* = −2.201)^∗^
			Unaffected	6.6 (1.8)	*P* = 0.015 (*Z* = −2.433)^∗^	5.5 (1.1)	*P* = 0.028 (*Z* = −2.201)^∗^
		Healthy controls		5.1 (0.7)			
			Dominant				
			Nondominant	7.4 (1.3)			


Action planning (%) Mean (SD)	Stick Test	CP patients	Unaffected	86.3 (15.1)	*P* = 0.003 (*Z* = −2.932)^∗^	93.3 (11.2)	*P* = 0.034 (*Z* = −2.121)^∗^
		Healthy controls	Dominant	100.0 (0)			
	Hammer Test	CP patients	Unaffected	58.3 (33.6)	*P* = 0.003 (*Z* = −2.990)^∗^	75.8 (20.2)	*P* = 0.042 (*Z* = −2.032)^∗^
		Healthy controls	Dominant	94.7 (7.7)			

CP: cerebral palsy; s: seconds; SD: standard deviation; %: percentage. ^∗^Statistically significant (*P* < 0.05).
